# Quantum Dynamics and Non-Local Effects Behind Ion Transition States during Permeation in Membrane Channel Proteins

**DOI:** 10.3390/e20080558

**Published:** 2018-07-27

**Authors:** Johann Summhammer, Georg Sulyok, Gustav Bernroider

**Affiliations:** 1 Institute of Atomic and Subatomic Physics, TU Wien, Stadionallee 2, 1020 Vienna, Austria; 2 Department of Biosciences, University of Salzburg, 5020 Salzburg, Austria

**Keywords:** ion channels, selectivity filter, quantum mechanics, non-linear Schrödinger model, biological quantum decoherence

## Abstract

We present a comparison of a classical and a quantum mechanical calculation of the motion of K^+^ ions in the highly conserved KcsA selectivity filter motive of voltage gated ion channels. We first show that the de Broglie wavelength of thermal ions is not much smaller than the periodic structure of Coulomb potentials in the nano-pore model of the selectivity filter. This implies that an ion may no longer be viewed to be at one exact position at a given time but can better be described by a quantum mechanical wave function. Based on first principle methods, we demonstrate solutions of a non-linear Schrödinger model that provide insight into the role of short-lived (~1 ps) coherent ion transition states and attribute an important role to subsequent decoherence and the associated quantum to classical transition for permeating ions. It is found that short coherences are not just beneficial but also necessary to explain the fast-directed permeation of ions through the potential barriers of the filter. Certain aspects of quantum dynamics and non-local effects appear to be indispensable to resolve the discrepancy between potential barrier height, as reported from classical thermodynamics, and experimentally observed transition rates of ions through channel proteins.

## 1. Introduction

**Figure 1:**
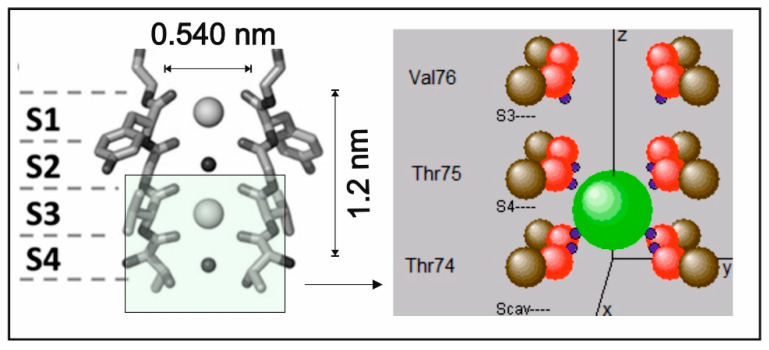
A section through the tetrameric KcsA filter motive, showing a sketch of two transmembrane helices for binding sites S4–S1, with two ions and two waters molecules (left). On the right, a window (insert) for atomic locations of the filter lining during the passage of a K^+^ ion (green) from S4 to S3 is sketched. The carbon atoms (brown) of the carbonyl groups are situated at the corners of a square (including all four backbone strands). The charge (blue) of oxygen atoms (in red) is partly contained in the center of the atom and partly within a point location slightly outside the oxygen. As these charges are drawn towards the central axis, they represent the effective charge center of the lone pair electrons (shown in blue). The size of atoms and the K^+^ ion on the right are drawn to scale approximately.

Selective translocation of ions bound to charges across the plasma membrane of cells provides the physical background for the generation and propagation of electrical membrane signals in excitable cells, particularly in nerve cells. The molecules organizing this translocation are provided by membrane-integrated channel proteins, which control the access of ions to permeation (“gating”) in response to changes in transmembrane potentials (“voltage-gating”) and allow very fast ion conduction without loss of selectivity towards certain ion species [1,2]. An unprecedented series of studies of these proteins has been initiated after the elucidation of the atomic resolution crystal structure of the prototypic *S. lividans* K^+^ channel by MacKinnon et al. [3]. It turned out that the critical domain of the protein that can combine fast transduction close to the diffusion limit with selective preference for the intrinsic ion species is provided by the narrow selectivity filter (SF) of the protein [4]. In particular, an evolutionary highly conserved sequence of amino acids, the TVGYG (Thr75, Val76, Gly77, Tyr78, Gly79) motive lining the filter region, allows for an inward orientation of backbone carbonyls with oxygen-bound lone pair electrons interacting with the positively charged alkali ions (see Figure 1). This delicate arrangement involving glycine (Gly79, Gly77) residues serving as “surrogate D-amino acids” [5] can offer a unique “interaction topology”, mimicking the ions’ hydration shells prior to entering the filter pore. The interactions are realized by short-range attractive (filter atoms) and repulsive (between ion) Coulombic forces [2,6].

The initial picture of ion conduction states was built on an alternating sequence of ions and water molecules (e.g., KwKw) passing through the filter with four equally spaced ion binding sites (labeled as S0–S4, as seen from the extra to the intracellular side) [4]. However, in the course of molecular dynamics (MD) studies this view has become considerably relaxed (e.g., by the observation that different ion permeation mechanisms may coexist energetically [7], pairwise water-ion hopping mechanisms can occur [8], the selectivity filter by itself could play a role in gating [9], and fast permeation involves a direct Coulombic “knock-on” without intermittent water [10]). Even more important are observations from MD studies calculating potentials of mean force (PMF) within the filter, which demonstrate that the potential barriers for ion translocation are simply too high (>5 kT at 300 K) to be in line with experimental conductance as predicted by the Nernst–Planck equation [11]. This seems to be a reflection of an enduring problem within the structure-function relations of purely classical MD simulations at the atomic scale and marks the point where the intention of our present contribution becomes significant.

Generally, the short range Coulombic forces coordinating the atoms in the filter reflect quantum-mechanical effects [2]. As argued before, this requires some quantum dynamics to account for the observed atomic behavior within their molecular environment [12,13,14]. In our previous work, we have suggested a role for a quantum physical description of ion motion through the selectivity domain of K^+^ channel proteins. In these studies, we suggested evidence that at least two important features behind ion permeation and gating dynamics can follow naturally if quantum properties are inserted into the underlying equations of motion. First, inserting quantum interference terms into the canonical version of action potential (AP) initiation can reproduce the fast onset characteristic of APs as seen in experimental recordings of cortical neurons [13]. Second, we have demonstrated evidence for different quantum oscillatory effects within the filter’s atomic environment, which discriminate intrinsic (e.g., K^+^) from extrinsic (e.g., Na^+^) filter occupations in K^+^-type channels [14].

In the present paper, we go beyond classical MD simulations by treating the motion of the ion itself in a quantum mechanical (QM) context. Because the de Broglie wavelength of thermal ions at 310 K (~0.025 nm) amounts to up to 10% of the spacing in the periodic structure of Coulomb potentials (~0.3 nm) in the nano-pore model of the selectivity filter, the ions wave packet is found to spread out over a certain region. The associated wave dynamics have a coherent short-lived and significant effect on the Coulomb interaction with the surrounding carbonyl charges. Based on first principle methods and solving a non-linear version of the Schrödinger equation, we find that the quantum trajectory of an ion through the filter is accompanied by different time-dependent phase velocities that can exert a favorable effect on the passage of ions through the confining potential landscape of the filter. We suggest that this observation from a combined QM-MD calculation can possibly explain fast conductance without compromising selectivity in the filter of ion channels. We shall discuss the way this favorable effect is exerted and to what extent it lowers the effective potential barriers for ion translocations. Phrased loosely, it is found that the front part of the particle wave function paves the path for the remaining wave components to “sneak through” the open doors of the confining potentials. Due to the involved barriers and masses, this process is different from “particle tunneling” (although it is naturally considered in the solution of the Schrödinger equation calculated with the Crank–Nicolson formalism [15]). Yet, another finding is remarkable within the context of the enduring debate about quantum coherence times in biological organizations: The quantum characteristic for the present effect not just builds on but also requires very short decoherence times (around 1 ps), a scale that is well within the expected range at biological temperatures [16].

Finally, it should be mentioned why we deal with a “non-local effect”, as expressed in the title of this paper: Most frequently, the term “non-locality” in QM states refers to a spatial separation between observables preserving a QM correlation (entanglement) between different modes (i.e., pertaining to different sub-systems behaving as one system). Here, we deal with only one technical QM mode or system (i.e., the ion). However, the present finding, that a short and coherent “spread” or “smear” over space of the particle’s mass-bounded charge, according to its QM wave function, can have a strong effect on the dynamic behavior within its environmental potentials implies a functional role for a “non-local property” of a single mode (or system). 

## 2. Methods

Our intention was to observe the K^+^ ion during the transition from site S4 to site S3 in the selectivity filter of the KcsA channel (Figure 1). Therefore, the simulation included the carbonyl groups of Thr74, Thr75, and Val76, as shown in Figure 1 (right). The backbone carbons were positioned at the widely used coordinates of Guidoni and Garofoli [17,18]. The carbonyl oxygen–carbon bond was set initially to 0.123 nm bond length and, in the force-free, unperturbed situation, pointed straight to the central axis (the *z*-axis of the coordinate system). Oxygen atoms were allowed to oscillate within horizontal and vertical bending modes, excluding stretching. The effective spring constant and the damping factor of this oscillation were adjusted to the values of typical thermal frequencies in the range of a few THz and to the expected dissipation of vibrational energy into the protein backbone structure after a few oscillation periods. Although considered in the implementation of the program, the short time interval in the present study did not necessitate setting thermal random kicks from backbone atoms to carbonyl atoms (Table A1). The degrees of freedom for the motion of a single K^+^ ion were constrained to the central *z*-axis of the selectivity filter. This allowed us to implement all calculations on a quad-core computer within a reasonable processing time and does not influence or restrict the conclusions to be drawn from the present results.

The Coulomb type interaction potential between two particles located at *r*_1_, *r*_2_ and charges *q*_1_, *q*_2_, including a repulsion term with a characteristic distance *r_cut_* (the distance where the electron shells start to overlap), and *ε*_0_ the vacuum dielectric constant, is as follows:

(1) $$V\left(r_{1},r_{2}\right)=\frac{q_{1}q_{2}}{4\pi \varepsilon_{0}}\left(\frac{1}{\left|r_{1}-r_{2}\right|}+\mathrm{sgn}\left(q_{1}q_{2}\right)\frac{r_{cut}}{{\left(r_{1}-r_{2}\right)}^{2}}\right)$$

where “point-charges” are located at the center of the particle. K^+^ ions carry unit charge and carbonyl-bound C atoms carry partial charges, usually set to +0.38 units. We assigned two-point charges to oxygen atoms; one at the center of the atom, and the second one representing the effective charge center of the lone pair electrons coordinating the K^+^ ions and/or water dipoles. The partial charge relocation between the lone pairs and the central O positions was chosen as one of the dynamic variables that determines the depth of the ion-trapping potential (Table A1).

The classical part of the present MD simulation is based on Verlet’s algorithm applied to Lennard-Jones molecules [19,20]. The QM model applies to the motional behavior of the K^+^ ion particle waves and was obtained from a non-linear Schrödinger equation (NLSE) (see Equation (2)), with an initial Gaussian wave packet set to an adjustable width and an adjustable mean ion velocity along the *z*-axis of the filter. The range of these settings is given in Appendix A in Table A1. It is assumed that the wave packet experiences a potential at every instant of time *t*, which depends on the position of all other particles at this time. Together with the potential term in Equation (1), this can be described by the following NLSE:

(2) $$\left[-\frac{\hbar^{2}}{2m_{K}}\frac{d^{2}}{dz_{K}^{2}}+\sum_{i=1}^{36}V\left(r_{i}\left(\psi \left(r_{K},t\right)\right),r_{K}\right)+gz_{K}\right]\psi \left(r_{K},t\right)=i\hbar \frac{\partial }{\partial t}\psi \left(r_{K},t\right)$$

where *m_k_* denotes the mass of the K^+^ ion and *r_k_* its position vector along the *z*-axis of the filter. Due to the tetrameric lining of the observed motive (Figure 1), summation over the potential term runs over 12 backbone atomic positions for the carbon, oxygen, and lone pair centers within the Thr74, Thr75, and Val76 lining amino acids shown in Figure 1 (right). As the atomic positions *r_i_* change in time and are influenced by the position of other atoms, as well as by the probability distribution of the K^+^ ion, the situation entails non-linearity in the Schrödinger equation (see also Appendix B). In the above Equation (2), this functional dependence of *r_i_* on *ψ* is explicitly indicated. The linear gradient “*g*” expresses the transmembrane electric potential and *z_k_* the z-component of the vector *r_k_*. The parameters that determine the shape and scaling of interaction potentials (i.e., the geometrical embedding) were adjusted to previous models of the KcsA channel [8,11]. This implied initial values for $r_{cut}$ of 0.13 nm, with the charge separation distance of the lone pair electrons from an oxygen center being 1.4 times the radius of the oxygen atom, leading to an average of 0.0825 nm from the oxygen atom’s center. The partial charges of an oxygen atom were split to contain a fraction of 30% at central locations and 70% in the lone pair charge point location (Table A1).

It should be noted that the NLSE given by Equation (2) restricts the motion of the K^+^ ion to the *z*-axis and does not include an expansion of the wave packet perpendicular to this axis. This restriction was necessary to keep the computational time within reasonable limits. In addition, the narrow extension and the symmetry of the filter lining in the pore cause sideways forces to mostly cancel each other along the filter’s *z*-axis. For the further formal description, this implies that the position vector $r_{K}$ of the K^+^ ion is essentially given by its *z*-component $z_{K}$, because its *x*- and *y*-components are always zero.

Prior to solving the SE given by Equation (2), we have to take into account the experience of forces by the surrounding carbonyl C and O atoms due to the interacting K^+^ wave. At this stage, we assume the backbone C atoms to be rigid (see Section 4) but allow for two bending modes of O atoms while keeping the CO distance constant. The differential *dz* along the *z*-axis of the wave packet is then found to exert a differential force $d{\vec{f}}_{i}$ as follows:

(3) $$d{\vec{f}}_{i}=-\vec{\nabla }V_{i}{\left|\psi \left(r_{K},t\right)\right|}^{2}dz\;=\frac{q_{i}q_{K}}{4\pi \varepsilon_{0}}\left(\frac{1}{{\left(r_{i}-r_{K}\right)}^{3}}+\mathrm{sgn}\left(q_{i}q_{K}\right)\frac{2r_{cut}}{{\left(r_{i}-r_{K}\right)}^{4}}\right)\left(r_{i}-r_{K}\right){\left|\psi \left(r_{K},t\right)\right|}^{2}dz.$$

The total force ${\vec{F}}_{i}$ acting from K^+^ on this O atom is then obtained by integrating over the range defined along *z* (additional forces acting on this O atom are a restoring force and a decelerating force, as well as attraction/repulsion of the surrounding C and O atoms, see Appendix C.) This will subsequently change the locations *r_i_* of the O atoms and thereby the potential term in the SE acting back on the evolution of the wave packet. The effect of Equation (3) introduces a non-linearity into the SE as shown in Equation (2). The resulting non-linear Schrödinger equation (NLSE) is formally similar but causally different from the description of Bose–Einstein condensation (BEC) at ultra-cold temperatures [21]. This is because under BEC conditions, the probability distribution of the wave function enters into the Hamiltonian itself, while in our case it is the effect integrated over time, as shown in Appendix B. We solved Equation (2) together with Equation (3) in very small-time steps by the Crank-Nicolson method [15] to keep track of the QM phase factor but sampled the positional changes of the O atoms in larger time steps (values given in Appendix A, additional explanations on the NLSE derivation in Appendix B).

**Figure 2:**
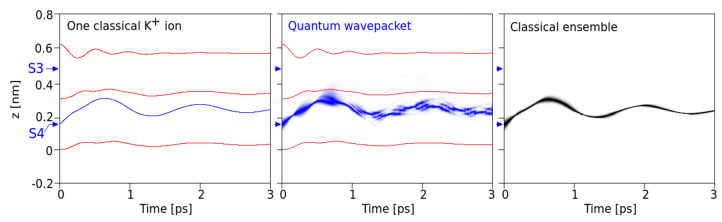
Single ions and the classical ensemble: Comparison of the evolution (within 3 ps time) of a single classical K^+^ ion (**left**, blue curve) with initial velocity of 300 m/s at the minimum of site S4 with a quantum mechanical wave packet of minimum uncertainty of this ion (**middle**). The red lines are the *z*-coordinates of the carbonyl oxygen atoms. Middle: Probability density from a quantum mechanical (QM) calculation along the *z*-axis of the wave packet as a function of time (intensity of blue reflects higher probability densities). The initial full width of this density is 0.05 nm (at 1/e). **Right**: Classical probability density of finding an ion from the ensemble of 10^4^ ions at the given *z*-coordinate as a function of time.

**Figure 3:**
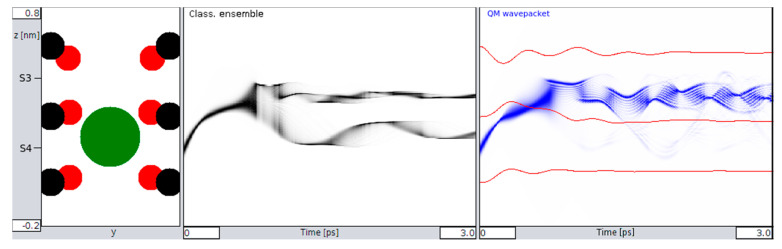
Transition behavior between S4 and S3 (**left** insert) for a classical ensemble (**middle**) and the simulated QM wave packet (**right**), with shades of black and blue coding normalized probability densities for location and time. Red lines (**right**) are again the *z*-coordinates of carbonyl oxygens. Note: whereas the classical ensemble splits after around 0.8 ps (**middle**), the QM distribution goes beyond the barrier to S3 almost completely (**right**).

**Figure 4:**
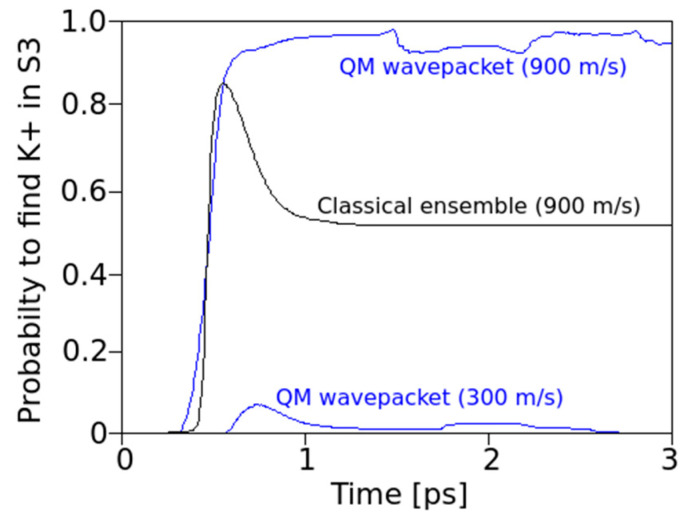
Time-dependent probabilities to find an ion in S3, when the ion was implanted into S4 with different mean onset velocities (900 m/s blue top for the QM wave packet, black for the classical ensemble) and at 300 m/s for the QM wave packet (with some probability <0.1 to cross over to S4). At this initial velocity of 300 m/s, the classical particles do not cross to S3. Note: most classical particles with 900 m/s are in S3 after 0.5 ps but eventually about 45% return to S4 due to oxygen charge derived forces (the spring that returns these ions to equilibrium positions with vibrations around 3 THz, see Figure 2). The QM wave exhibits a small but remaining probability (<10%) of returning to S4.

**Figure 5:**
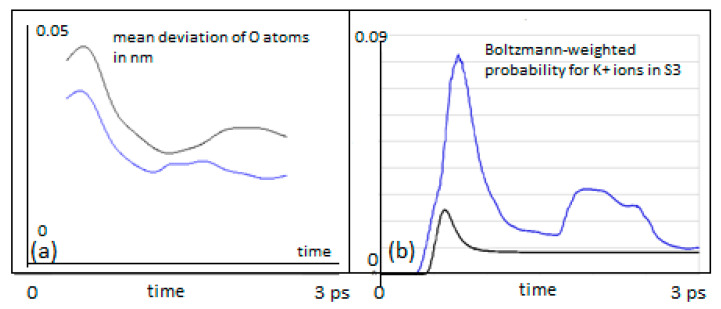
(**a**) Mean deviation of Tyr75 carbonyl oxygens from their equilibrium positions, while a K^+^ is moving past from location S4 to S3 (in nm). The classical particles are in black, QM wave packets in blue; (**b**) Probability for a K^+^ ion to be found in S3, setting out from S4 with mean velocities between 100 m/s and 900 m/s, weighted according a Boltzmann velocity distribution at 310 K (blue QM, black classical).

We simulated the behavior of classical ensembles to compare with the above-described quantum behavior in Figure 2, Figure 3, Figure 4 and Figure 5. In these simulations, classical particles were set into motion at 10^2^ different starting positions. The positions were generated equidistantly within three times the full 1/e-width of the initial quantum wave packet and weighted with the probability density of this packet. At each initial location 10^2^, particles were set into motion with velocities, again sampled equidistantly from within three times the full 1/e-width of the initial Gaussian momentum distribution of the QM wave packet.

The numerical implementation of the present methods was designed to offer an interactive control window that allowed us to change the settings given in the Appendix A for path integration according to Equations (2) and (3), and it delivered the following graphs for time-dependent wave functions and probability plots (Figure 2 and Figure 3).

## 3. Results

### 3.1. Classical versus QM Motion

First, our intention focused on the comparison between the classical standard MD setting and the quantum mechanical version under the same interaction potentials between all constituents and the same initial position and velocity distribution of the ion (Figure 2). This yielded comparable results under situations where the K^+^ ion is coordinated at a specific site (e.g., S4) and oscillates within this site due to its thermal energy. In the QM version, the wave packet is placed at the minimum of the potential of this site (at *z* = 0.15 nm) and assigned a mean velocity corresponding to a kinetic energy sufficiently below the potential barrier to the next site. For the examples shown, we have chosen *v*_0_ = 300 m/s. As can be seen from Figure 2, under identical initial conditions at time *t* = 0, the temporal behavior of a single classical ion (left) is similar to the behavior of the QM wave packet, with the same frequency (around 900 GHz) and amplitudes. This similarity becomes even more striking in a comparison of the single wave packet with a classical ensemble of ions computed for 10^4^ particles under an identical initial position and velocity distribution as in the QM version (Figure 2, right). 

However, even at the initially relatively low kinetic energy levels of a K^+^ ion, the QM wave shows a non-vanishing probability that the ion could make a transition to site S3 in the filter (as seen around <1 ps after onset and in more detail in Figure 4, bottom). This observation is particularly interesting as it occurs within just one picosecond (i.e., well within the expected decoherence time due to thermal noise from protein backbone atoms transmitted to carbonyl atoms). 

### 3.2. Transition Behavior: Classical versus QM Evolution

At the onset of the transition between sites S4 and S3 (i.e., when the initial velocity of the K^+^ ion approaches the values needed to cross the potential barrier between these two sites), the QM wave packet and the classical ensemble start to behave quite differently. Figure 3 captures an example for a velocity of *v*_0_ = 900 m/s of the ion. At the boundary to S3, the classical ensemble is found to split into roughly ½ (Figure 3, middle), whereas the QM behavior shows that the wave packet manages to cross the barrier with almost all of its location probability (Figure 3, right). Following these initial characteristics, there are also subsequent differences: The splitting in the classical picture remains unchanged during the observed time interval. Ions that have crossed to S3 remain in S3, and ions that did not cross remain in S4.

Despite crossing the potential to S3, the QM wave packet retains a probability of up to 10% to return to location S4 (Figure 4 below). This effect may look inconspicuous at first glance, but in fact, it marks a highly relevant difference between a classical and a quantum mechanical behavior of the moving ion (see Section 4). The passage of “classical ions” through the close coordination distances (in the range of 0.27 nm [17]) provided by the Thr75 oxygen lone pairs, directs these charges to a point towards the center of the ion. Along this view, the ion “drags” a potential valley along its path. In the quantum situation, however, the direction of these forces can split up into different components directed towards a delocalized charge-probability distribution along some distance in the *z*-direction. One effect to be expected from this interference is that, while the front part of the distribution with the fast-moving momentum components attracts these oxygens towards it, the slower moving “tails” can follow without having to overcome the high barriers.

A signature of this possibility can be seen from the mean deviation of the Tyr75 carbonyl oxygens from their equilibrium as the K^+^ ion passes, shown in Figure 5 (left). The deviation depicted in this figure is permanently lower for the QM K^+^ ion as compared with the classical ensemble of ions.

### 3.3. Transition Behavior over a Range of Ion Velocities

The above findings relate to specific initial velocities. From a more general view, one has to take into account that an ion at a specific position (e.g., S4) will oscillate due to its thermal environment and may therefore take on a wide range of velocities. We have therefore performed a series of simulations for both the classical ensemble and the QM version for initial mean velocities between 100 m/s and 900 m/s. For this series of mean velocities (at steps of 100 m/s), the probability to locate the K^+^ ion in S3 was calculated (Figure 4) and the resulting curves were corrected by weights obtained from a Boltzmann distribution (which provides the probabilities for each of the initial mean velocities to occur at a temperature of 310 K). The sum of individual probabilities finally provides a Boltzmann-weighted distribution for the probability to find the QM ion at site S3 at a given instant of time (Figure 5b). We have chosen the same procedure for the weights of classical probabilities.

Figure 5b provides a summary of the results for the probabilities of finding a K^+^ ion in S3, setting out from a starting location at S4. As mentioned, the probabilities were Boltzmann weighted for 310 K and summed over velocities from 100 to 900 m/s in steps of 100 m/s.

The results shown in Figure 5b clearly demonstrate that the QM wave has an increased probability of crossing from S4 to S3 as compared with the classical particle, throughout the time interval studied and the range of initial velocities. During an early stage of the transitions (*t* < 1 ps), the QM probability for S3 is more than three times the classical probability. After a very short initial time (*t* < 0.5 ps), when the classical ion has hardly reached the barrier, a QM ion has already acquired a significant probability for having crossed this barrier. The QM particle also shows periodic interferences during the 3 ps time interval that are not apparent for a classical behavior with a tendency to return to S4 after 2.5 ps. The classical particle adopts a constant and low probability to transit after about 1 ps. In both cases, in the classical and in the QM situation, the transfer probability to S3 settles to about 10% after 3 ps. This seemingly low value is consistent with a “field-driven” and diffuse transfer through the selectivity filter with alternations between site halts involving oscillations in thermal equilibration and subsequent “hopping” to the next site, a situation that is well predicted by previous MD studies (e.g., [9]). In the “conductive state” of the filter, the ion would naturally transit to the subsequent site S2 along the conduction path (“z” in the filter model). In the “non-conductive filter state”, alternating site changes between the configurations 1,3 and 2,4 can occur, which includes a return path from S3 to S4 [3,4,9]. As in the present study, the focus was laid on a single site transition within the conduction cycle from S4 to S3; the coordinating carbonyl groups from subsequent amino acids beyond Val76 were not included in the simulation. Subsequent studies will gradually have to involve the entire conduction path to account for all interaction terms in the guidance of the ion. However, as the force from attractive potentials exerting from distant (e.g., S2) carbonyl cages drops with 1/Δ*r*^2^ (Equation (1)) and these distances can be expected to have a lower bound around ∆*r* ≥ 0.6nm, the effect on S4–S3 transitions from more distant oxygens can be expected to be small (in addition, the expected intermittent water dipoles will exert a damping effect on these forces). We provide further comments on this situation in Section 4.

Our observations can shed light on the question of a decoherence function during ion transitions (see Section 4). We find that for a QM wave description of an ion within the potential landscape of the filter atoms, short coherence times are beneficial for the observed high conductivities. If coherence time is short (i.e., not much longer than around 1 ps), the wave packet of the ion can cross the barrier much easier (the peak in Figure 5b) due to its quantum nature as compared to a classical ion. The following loss of coherence, however, is equally important because having crossed the barrier, decoherence will eliminate QM interferences, and the particle starts to adopt classical behavior, which avoids the undesired return to S4. The question of coherence, of course, deserves additional attention, as given in the following section.

## 4. Discussion

We have investigated the motional behavior of a single K^+^ ion between two transition sites in the nano-pore selectivity filter motive of the KcsA channel from two different perspectives: A quantum mechanical simulation implemented by a non-linear version of the Schrödinger equation and a corresponding classical ensemble behavior under identical initial and interaction terms. The non-linear Schrödinger model (NLSE) integrates the solution of the wave equation into its interaction potentials, with all surrounding charges modulating the probability distribution of the wave at a given instance of time. This offers a kind of recursive approach, taking account of mutual interactions of confining Coulomb forces and the QM wave equation, a situation that seems more realistic than the calculation of potentials of mean force (PMF) from classical MD at the atomic scale. The methods were implemented in Java. An executable version is available upon request, as well as a source citation agreement (this requires prior installation of the Java Runtime Environment). 

First, our results provide a comparison between classical and QM implementations, which reveal a high degree of similarity in the overall, time-dependent behavior during a several pico-second time interval (Figure 2). The observed similarity is particularly obvious for the ensemble behavior of ions and certainly signals a high level of consistency of the implemented methods. Besides this similarity at the “caged” state, as shown in Figure 2, we also observe small but significant differences in the time evolution of QM and classical ions prior to transitions from S4 to S3 that bear the “seeds” for subsequent QM classical dissociations during the passage from one binding site to the next site. Following this initial situation, the site transition favored by higher onset velocities marks a clear difference between the behavior of the QM wave packet and the classical ensemble of ions. At a critical velocity of ions, the classical probability splits into ½ of its population, with 50% crossings to S3 and the rest remaining in S4 (Figure 3 and Figure 4). Within the same time, and under the same initial conditions, the QM packet, however, can cross to S3 almost completely. An increase in efficiency to cross the barriers of about 50% could be expected to increase permeation rates at larger scales by a similar amount (i.e., within a complete filter occupancy and including intermittent water molecules in the filter domain). Compared with previous reports about the discrepancy of calculated energetic barrier heights from PMF methods (between 5 and 10 kcal/mol) with observed and “effective permeation heights” as required by Nernst–Planck estimates of <~3 kcal/mol at 300 K [11], the ratio of this difference lays well within the range of permeation enhancement as predicted here. In other words, assigning a short (1 ps) coherent quantum mechanical property to the atoms motion interacting with the carbonyl derived forcefields can explain fast conduction speeds, whereas purely classical models cannot.

It must be granted that the present report focuses on a small and short time-scaled view on filter dynamics. This may somehow insufficiently sample the more complete conformational pattern underlying filter conduction states. However, the main findings of the present study strongly suggest that a small scaled and ultra-short-lived quantum state of the permeating ion is not just sufficient but also indispensable to explain fast conduction without compromising selectivity in the filter. The observations demonstrated in Figure 4 and Figure 5 indicate that due to the dispersion of the wave function of a “quantum ion”, the coordinating Coulomb forces from surrounding oxygen charges become dissociated to different parts of the wave function. This effect reduces the effective barrier that the ion has to cross. In a metaphorical view, it looks like the faster front part of the particle wave can open the door to the barrier, allowing the slower tail parts to sneak through this door. Due to the engaged height and width of the barrier, this effect is not typical “leap-frogging” (passing over), nor “quantum-tunneling”, although there is some formal resemblance to the latter. The resemblance is that the potential energy of the separating barrier is also a function of the state variable of the system. But, as opposed to tunneling, the system actually “manipulates” the barrier to be able to cross it. It is perhaps best described as “*quantum sneaking*” through potential barriers.

The present study takes into account physiological temperatures with respect to ion motion and velocities. At this stage, the effects of these temperatures on thermally induced protein backbone and carbonyl vibrations are not included. Our numerical implementations do offer the extension to thermal carbonyl atomic motions during the temporal evolution of the K^+^ wave packet. We presently implement these fluctuations by repetitions of K^+^ evolutions during time-varying random fluctuations of the surrounding O and C atoms. We expect, however, that the main findings reported here about the difference between a classical ensemble and the K^+^ wave packet will remain largely resistant to these thermal vibrations. The reason is that the main effect found here is due to the spatial dispersion of the QM wave packet, which in turn dynamically spreads the interacting force directions of the surrounding and coordinating charges. In the pure classical case, these forces would permanently be directed towards the center of the moving ion. It is just this distinction that allows for what we called “quantum sneaking” in the discussion above.

Finally, our observations render an important role for decoherence and the quantum to classical transition as predicted in an earlier paper by one of the authors [22]. Fast decoherence of the ions wave function after about 1 ps, a time when almost all of its probability distribution has penetrated the barrier, leads to classical behavior, which can be seen to avoid the return to the previous location. So, decoherence actually “guides” the particle into one direction in the filter, and an oscillation between quantum and classical states cooperate in a directed transport through the potential landscape of the filter.

A possible role of the important and unique QM interaction, the environment-induced, dynamical destruction of quantum coherence deserves some further remarks in the present context. It was not our intention to study decoherence in our simulation explicitly, and we did not include the scattering elements and processes that induce decoherence in the evolving wave packet. In some previous work, we and a co-author of this group (V. Salari) have provided a list of scattering sources and interacting scattering events applicable to the same atomic configuration and dynamics of the KcsA filter model as used in the present study [14,16,23]. The results of these studies suggest that we can expect decoherence times for K^+^ ions in the filter model around one or a few pico-seconds at warm temperatures.

The intention of the present simulation was more focused on a potential functional role of decoherence during ion permeation by implementing a comparison between quantum and classical motions. As we can expect that decoherence of the QM wave packet will lead to a certain resemblance with a classical behavior in the course of time, comparisons as those shown in Figure 4 and Figure 5b can give us some inferential information about the time and the role of decoherence. It turns out, that short decoherence times, exactly within the range of the predictions mentioned from the scattering studies above (i.e., around 1 ps), could play a highly beneficial role for the successful transition from S4 to S3. A transition to classical behavior due to decoherence after this time would actually “stabilize” the ion’s location at S3 once it has reached this site. The original transition probability would still be at the level of the high QM initial transfer probability. We therefore conjecture that short coherent QM states, in the range of a few ps, are of an advantage for the observed high ion transfer rates without compromising ion coordination. Taken together, we suggest that the quantum dynamics behind the ion motion in the filter open the door through confining potentials, and decoherence guides the moving atoms through the specific path offered by the selectivity filter of channel proteins. To the best of our knowledge, this is one of the first reports about a decisive role of quantum decoherence for an ancient and highly conserved mechanism of membrane signaling in biology.

## Appendix A

**Table A1:** Constants and parameter settings.

Charge of the K^+^ ion	+1 *q*_0_ (*q*_0 …_ unit charge)
Charge of the carbon of a CO-group	+0.38 *q*_0_
Charge of the oxygen of a CO-group	−0.38 *q*_0_
Distance C–O of a CO-group	0.123 nm
*z*-coordinate of the CO carbons at Thr74	0 nm (by definition)
*z*-coordinate of the CO carbons at Thr75	0.30 nm
*z*-coordinate of the CO carbons at Val76	0.62 nm
Distance of the CO carbon atoms from axis of selectivity filter (*z*-axis)	0.38 nm
Stiffness of bending of the O atom around the C atom in a CO-group	30°/k_B_T
Damping constant of rotational vibrations of O atoms	1 × 10^−13^ kg/m
Positions of oxygen atoms at *t* = 0	equilibrium positions ^1^
Velocity of oxygen atoms at *t* = 0	0
Distance of lone pairs charge from the center of the O atom	1.4 *r*_0_ (=0.0825 nm)
Percentage of O partial charge in lone pairs	70%
*r_cut_*	0.13 nm
Thermal random kicks from backbone to carbonyls	None
Linear potential drop along the axis of the selectivity filter	−100 mV/nm
Initial position of K^+^ ion	0.15 nm ^2^
Initial mean velocity of K^+^ ion wavepacket or classical ensemble	varied between 100 m/s and 1200 m/s
Full width of wavepacket (1/e-width)	0.05 nm ^3^
Time step for the classical calculations with Verlet algorithm	1 fs
Time step for the quantum mechanical calculations with Crank–Nicolson algorithm	0.003 fs
Time step for sampling positional changes of O atoms due to the K^+^ force	6 fs

^1^ C–O perpendicular to axis of selectivity filter and pointing to this axis. ^2^ This is approximately the middle of site S4. ^3^ This width entails a velocity spread (1/e-full width) of ±65 m/s. Making the wave packet much narrower would give velocity spreads on the order of the thermal mean velocity of a K^+^ ion. Making it much wider would bring it beyond the width of the ground state of the harmonic oscillator to which a site potential can be approximated.

## Appendix B. How the Schrödinger Equation Becomes Nonlinear

The standard Schrödinger equation (SE) of the K^+^ ion has an apparently linear form:

$$\left[-\frac{\hbar^{2}}{2m_{K}}\frac{d^{2}}{dz_{K}^{2}}+\overset{36}{\underset{i=1}{\sum }}V\left(r_{i},r_{K}\right)+gz_{K}\right]\psi \left(r_{K},t\right)=i\hbar \frac{\partial }{\partial t}\psi \left(r_{K},t\right)$$

The potential term of this equation contains the position vectors $r_{i}$. While the $r_{i}$ of the carbon atoms of the carbonyl groups are fixed in space, the $r_{i}$ of the oxygen atoms depend on time. In our calculations, each oxygen atom can rotate around the two orthogonal axes of a polar coordinate system with respect to the C atom of the carbonyl group, but the bond length is kept constant. An O atom is subject to the following forces: a restoring force, which is proportional to the angular elongation of the O atom from its unperturbed position; a dissipative force, which is proportional to its momentary velocity (see Appendix C); Coulomb forces from neighboring O and C atoms; and the force from the K^+^ ion, which depends on the quantum mechanical probability distribution of the K^+^ ion at that moment of time. This latter force introduces a non-linear aspect into the Schrödinger equation:

The time-dependent position vector $r_{i}\left(t\right)$ of the *i*-th O atom is given by the following relation

$$r_{i}\left(t\right)=r_{i}\left(0\right)+v_{i}\left(0\right)\cdot t+\int_{0}^{t}\left[\int_{0}^{t^{\prime }}{\ddot{r}}_{i}\left(t^{\prime \prime }\right)dt^{\prime \prime }\right]dt\prime$$

where $r_{i}\left(0\right)$ and $v_{i}\left(0\right)$ are the O atom’s position and velocity at time t = 0 and ${\ddot{r}}_{i}\left(t\right)$ is the acceleration at time t. By Newton’s second law, this acceleration is the quotient of the force acting on the atom, and the atom’s mass $m_{O}$, as follows:

$${\ddot{r}}_{i}\left(t\right)=\frac{{\vec{F}}_{i}\left(t\right)}{m_{O}}+\frac{{\vec{F}}_{other}}{m_{O}}=\frac{1}{m_{O}}\int_{z_{min}}^{z_{max}}df_{i}\left(z\right)+\frac{{\vec{F}}_{other}}{m_{O}},$$

where ${\vec{F}}_{i}\left(t\right)$ is the force due to the quantum mechanical probability distribution of the K^+^ ion and ${\vec{F}}_{other}$ subsumes all other forces acting on the atom.

With the differential force element $df_{i}$ from Equation (3) this acceleration becomes

$${\ddot{r}}_{i}\left(t\right)=\frac{q_{i}q_{K}}{4\pi \varepsilon_{0}m_{O}}\int_{z_{min}}^{z_{max}}\left(\frac{1}{{\left(r_{i}\left(t\right)-r_{K}\right)}^{3}}-\frac{2r_{cut}}{{\left(r_{i}\left(t\right)-r_{K}\right)}^{4}}\right)\left(r_{i}\left(t\right)-r_{K}\right){\left|\psi \left(r_{K},t\right)\right|}^{2}dz+\frac{{\vec{F}}_{other}}{m_{O}}.$$

Because the position of the K^+^ ion, $r_{K}$, is always on the *z*-axis, the integration extends over the whole range of the wave packet. For the present “cut-off” in our model, the wave packet has appreciable values only between the limits of $z_{min}=-0.2\;\mathrm{nm}$ and $z_{max}=0.8\;\mathrm{nm}$. From the above equations, one can see that the position of an O atom at a given moment is determined by the entire history of the spatial spread of the wave packet of the K^+^ ion. As this position determines the potential to which the K^+^ ion is exposed at that instant of time, it introduces the non-linear term into the Schrödinger equation as given by Equation (2).

## Appendix C. Restoring and Dissipating Forces on Carbonyl O Atoms

Generally, carbonyl O atoms of the filter lining can oscillate in two directions similar to a two-dimensional pendulum around the carbonyl C atom. This movement involves a damping term, which leads to a dissipation of energy into the protein backbone and to intermittent water molecules. Here, we model the restoring forces in the following way:

For an O atom out of its equilibrium position, which is defined by its position in the absence of any forces from other atoms and charges, the torque for a return to equilibration in the present model is given by the following:

$${\vec{T}}_{r}=-c\vec{a},$$

where c is an adjustable stiffness constant and $\vec{\alpha }$ is the two-dimensional vector angle on the unit sphere, specifying angular distance and direction from the equilibrium position. This torque can be translated into a restoring force acting on the O atom. Our choice in the present simulation was to adjust the constant “c” so that off-equilibrium angles of 30° are associated with an energy of $k_{B}T$ (see Table A1), as this gave the typical values for the expected vibrational frequencies.

The damping force was assumed to be characterized by a torque as follows:

$${\vec{T}}_{d}=-b\vec{\omega },$$

where b is a friction constant and $\vec{\omega }$ represents the angular velocity of the O atom at a given instant of time. This torque accounts for a force, which eventually slows down the motion of the O atom and thereby reduces its kinetic energy. In turn, this energy can be assumed to be lost into the protein backbone, water molecules, or other elements not explicitly modeled in the present study.
